# Tracheomalacia Treatment Using a Large-Diameter, Custom-Made Airway Stent in a Case with Mounier-Kuhn Syndrome

**DOI:** 10.1155/2014/910135

**Published:** 2014-09-08

**Authors:** Cengiz Özdemir, Sinem Nedime Sökücü, Levent Karasulu, Sedat Altın, Levent Dalar

**Affiliations:** ^1^Yedikule Chest Disease and Thoracic Surgery Research and Training Hospital, 34760 Istanbul, Turkey; ^2^Department of Pulmonary Medicine, School of Medicine, Istanbul Bilim University, 34394 Istanbul, Turkey

## Abstract

Mounier-Kuhn Syndrome (MKS) is a rare congenital disease that presents with abnormal enlargement in the central airways. In MKS, tracheomegaly is accompanied by difficulty in expelling recurrent lung infections and bronchiectasia. We presented a patient with MKS where commercially made stents were inadequate for stabilization and a custom-made, self-expandable metallic stent with a diameter of 28 mm and length of 100 mm was used. Chest pain that was thought to develop due to the stent and that disappeared after stent removal may be considered the main complication leading to stent removal. Continuous positive airway pressure therapy (CPAP) therapy was planned for the control of symptoms, which re-emerged after stent removal. This case is presented as an example that complications developing due to the stent as well as patient noncompliance may lead to stent removal, even when useful results are obtained from treatment of MKS.

## 1. Introduction

Mounier-Kuhn Syndrome (MKS) is a rare congenital disease that presents with abnormal enlargement in the central airways [1]. Enlargement in the central airways continues to the distal end of the trachea and main bronchus and the airways return to normal size after the fourth to fifth branching [2]. In MKS, tracheomegaly is accompanied by difficulty in expelling recurrent lung infections and bronchiectasia [3, 4].

In the involved airway segment, there is collapse at varying degrees at expiration and the disorder is among the congenital causes of tracheobronchomalacia (TBM) [5]. In cases where symptoms cannot be controlled with the treatment of infections and comorbid diseases, stent placement in the airways and surgical treatment (tracheobronchoplasty) options may also yield useful results [6].

In the present report, the placement of a large, custom-made tracheal stent and treatment outcome are discussed.

## 2. Case Report

**Figure 1:**
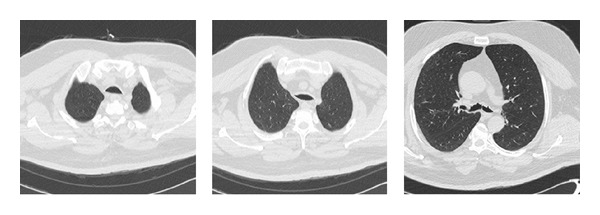
Thoracic computed tomography: in the airway segment starting from the proximal end of the trachea and extending to the main bronchi, an increase in the transverse diameter, a decrease in the anterior-posterior diameter, and diverticular irregularities in the level of the carina are observed.

A 58-year-old male patient presented with complaints of dyspnea on exertion, cough, sputum production, and snoring for the last 7 years. The patient stated that he was admitted to the hospital several times within the last 2 years due to the progression of these symptoms. He also received treatment for the diagnoses of hypertension, hypothyroiditis, and type II diabetes and used several types of inhaler treatment for dyspnea. Physical examination revealed that he was obese with a body mass index (BMI) of 41.8 and his respiratory sounds were rough with a long expiration time. However, examinations of his other systems were normal. His whole blood count, biochemical analysis, and thyroid function tests were unremarkable. On chest X-ray, enlargement in the transverse diameter of the trachea was detected; the spirometric test results are shown in Table 1. Arterial blood gas analysis in ambient air was also unremarkable. His thorax tomography is shown in Figure 1. Bronchoscopy revealed an enlarged transverse diameter of the trachea starting from the entrance and severe collapse was observed with respiration. The diameter of the trachea was measured to be 35 mm at the largest dimension. Enlargement and collapse continued in the right and left main bronchus. The diameter of the airways was normal at the distal end of the main bronchus. Polysomnography was also planned with the presumptive diagnosis of obstructive sleep apnea syndrome.

**Figure 2:**
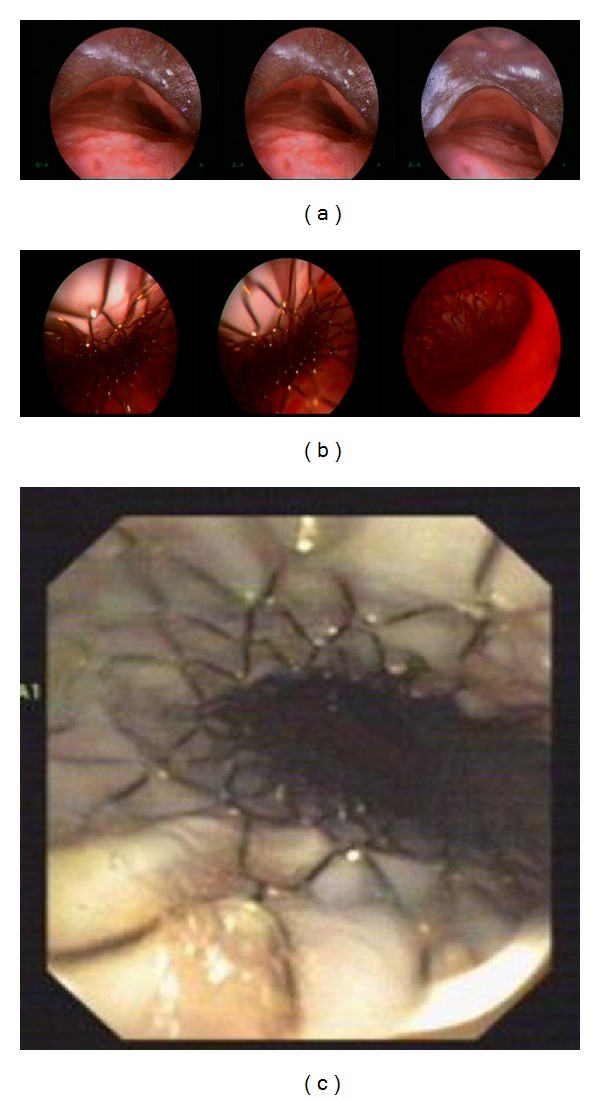
Pictures of stent during the rigid bronchoscopy procedure. Increase in trachea transverse diameter and severe malacia (a), trachea after stent placement (b), control visit at 1st month (c).

Stent placement was planned for airway stabilization. Because the diameter of the trachea increased excessively, it was not preferred to use stents with the largest diameter of 22 mm because of the high risk of migration. A custom-made, self-expandable metallic stent with a diameter of 28 mm and a length of 100 mm was ordered. The stent (Silmet; Novatech, La Ciotat, France) was produced within 3 weeks. Under general anesthesia, the stent was placed in the trachea using a rigid bronchoscope (Figures 2(a) and 2(b)). Following the procedure, airway stability was improved in the trachea. The patient was transferred to the hospital ward after staying for 24 h postoperatively in the intensive care unit. There was marked improvement in symptoms. The spirometry results before stent placement and at the first-month control visit are summarized in Table 1 and bronchoscopic view is given in Figure 2(c).

At the fourth-month control visit, the patient demanded that the stent be removed due to chest pain and inability to produce sputum, which he attributed to the stent. His cardiological examination was normal and the patient stated that he did not benefit from the treatment administered for the palliation of pain. Improvement in dyspnea and exercise capacity was still maintained. In bronchoscopy, minimal secretion was observed in the stent and the stent was observed to prevent collapse markedly during expiration. Although the patient was strongly advised about the benefit of the stent, he was not persuaded and the stent was removed under general anesthesia.

After the removal of the stent, the patient was still symptomatic. At the control visit, his BMI had increase up to 43.6 and polysomnography revealed an apnea-hypopnea index (AHI) of 25.6. However, the patient could not tolerate a nasal or an oronasal mask and stated that he could not accept this alternative treatment either.

## 3. Discussion

MKS was originally defined by Mounier-Kuhn [7]. The enlargement of the airways is accompanied by collapse in expiration, resulting in symptoms. Criteria have been defined to measure dilation by tomography [8]. A diameter of the trachea over 30 mm, right main bronchus over 20 mm, and the left main bronchus over 18 mm is considered dilation. The aim of treatment is to control symptoms by improving malacia. Therefore, treatment opt ions such as airway stabilization by stent placement and surgical methods are recommended in addition to the treatment of accompanying conditions. In TBM cases, airway stabilization using a stent or surgical methods is becoming more common at many centers [9–11].

This case was referred for evaluation upon continuation of symptoms despite medical treatment administered for COPD and TBM accompanying MKS. In bronchoscopy, dilation and malacia were found in a large airway segment involving the trachea and main bronchus that required evaluation for alternative treatment options other than medical treatment. In the present case, the tracheal segment with malacia was successfully stabilized with a custom-made stent. Although a stent is not used for the main bronchus, collapsibility of the main bronchus is partly prevented by the change in the respiration mechanics with the effect of maintenance of the tracheal lumen patency, stabilization, and also change of the tracheobronchial tree angulation. Because no complication was noted in the followup, improvement was found in his symptoms and respiratory function tests and stent placement in the segments with malacia in the main bronchi was not considered.

In TBM, airway stabilization is necessary for symptom control and prevention of complications such as frequent respiratory tract infections and the development of bronchiectasia. Using stents for airway stabilization is useful for symptom control in patients who cannot undergo surgical intervention [11]. In patients with MKS accompanied by severe TBM, experience regarding the success of airway stabilization is limited. Odell et al. reported that although MKS cases showed substantial improvement in quality of life, dyspnea scores, and symptoms after stent placement and tracheobronchoplasty, no significant change was found in forced expiratory volume in 1 s (FEV_1_) values [6]. In the present case, the FEV_1_ value was found to increase by 1500 mL after stent placement and this improvement was maintained at the four-month followup.

The most important problem that will be encountered in stent placement in cases with MKS is the lack of sufficiently large stents required for excessive dilation in the airway. Odell et al. reported that, in cases with MKS, migration was prevented by placing a silicon Y type stent in the airway [6]. However, in the present case, it was impossible to produce adequate stabilization in the airway with a Y stent because the trachea was dilated throughout its entire length with severe malacia. Considering that the stents that are currently available commercially would be inadequate for stabilization in this patient, a custom-made, self-expandable metallic stent with a diameter of 28 mm and length of 100 mm was used (Silmet; Novatech).

Surgery is the first line treatment in TBM. Stent application can help symptom palliation in patients in which surgery cannot be applied or refuse surgery [11]. Besides a stent is beneficial both for symptom control via airway stabilization and for the determination of patients who will benefit from surgical intervention [11]. Because our case refused the surgical treatment option, stent placement was attempted for symptom palliation.

Thus far, to the best of our knowledge, only one case report exists in which a custom-made large stent was used for tracheal dilation and malacia [12]. In a case with tracheomegaly associated with Marfan syndrome, due to malacia in the lower third segment of the trachea and main bronchi, a self-expandable, custom-made metallic stent with a diameter of 28 mm and a length of 69 mm was placed in the trachea and an 18-mm-diameter silicon stent was placed in the left and right main bronchi. Symptom improvement was maintained during 2 years of followup.

In airway stents, the formation of granulation tissue, mucostasis, infection, and migration are common complications [6, 12]; in some cases, it may be necessary to remove the stent for management of these complications. Probable complications that could develop in these patients and stent fractures could end up by the removal of the stent. For this reason, surgery should be kept in mind as a first line treatment in patients with TBM for both long term symptom palliation and not facing stent related complications. In the present case, no serious complication was observed except for chest pain attributed to the stent. Chest pain that was thought to develop due to the stent and that disappeared after stent removal may be considered the main complication leading to stent removal. Pressure exerted by the stent on a thinned tracheal wall was considered to be the main cause of retrosternal pain.

In cases with TBM, noninvasive positive pressure may help to decrease pulmonary resistance and respiratory work load and improve expiratory flow with airway stabilization and symptom control [5]. In the present case, continuous positive airway pressure therapy (CPAP), therapy was planned for the control of symptoms, which reemerged after stent removal, as well as for the treatment of obstructive sleep apnea syndrome. Because the patient did not accept CPAP either, its long-term effect on symptom palliation could not be evaluated.

## 4. Conclusion

In cases with severe TBM accompanied by MKS-associated tracheobronchomegalia, using large-diameter stents instead of conventional stents may be beneficial for airway stabilization and, hence, symptom control. Airway stabilization with stent can be the only option beside probable complications in cases in which traditional surgery cannot be applied. However, it should be considered that complications developing due to the stent as well as patient noncompliance may lead to stent removal, even when useful results are obtained from treatment.

## Figures and Tables

**Table 1:** Spirometric evaluation results before and after stent insertion.

	Before stent insertion	After stent insertion
FEV_1_ in liters (%)	1.43 (44.6%)	2.95 (111.7%)
FVC in liters (%)	2.92 (72.3%)	3.54 (114%)
FEV_1_/FVC	0.44	0.83

FEV_1_: forced expiratory volume in 1 s; FVC: forced vital capacity.
